# Surgical Management of a Large Chronic Prepatellar Bursitis: 2-Stage Technique

**DOI:** 10.1155/2020/3204014

**Published:** 2020-01-13

**Authors:** Sriskandarasa Senthilkumaran, Steven W. Hamilton

**Affiliations:** Department of Trauma and Orthopaedic Surgery, Woodend General Hospital, Aberdeen AB15 6XS, UK

## Abstract

Treatment of a large chronic prepatellar bursitis can be difficult to manage surgically because of a high rate of local complications and a significant chance of recurrence. We present a 2-stage technique using negative pressure dressings which produced a good outcome with no recurrence at one year after surgery.

## 1. Introduction

Inflammation of the prepatellar bursa is a common condition, especially in males. It is typically caused by repetitive injury and often seen in patients whose occupation involves kneeling. It is referred to by various eponymous names such as clergyman's knee and housemaid's knee. Acute inflammation can settle with the well-established nonoperative treatment algorithm of rest, ice, activity limitation, and anti-inflammatories [1].

Inflammation can however become chronic with the swelling becoming large and problematic. Surgical intervention is reserved for severe refractory cases. Open and endoscopic bursectomy procedures have been described [2–4]. Most of the techniques described in the literature are focused on septic prepatellar and olecranon bursitis. Recurrence rates have been reported as high as 20%. Complications of the open approach include wound haematoma, scar tenderness, damage to the infrapatellar branch of the saphenous nerve, seroma formation, and skin necrosis [5–7]. Endoscopic procedures are performed through multiple portals and can be effective in terms of postoperative recovery and cosmesis. However, the complication of recurrence remains due to inadequate excision of the bursal tissue [8]. There is also a risk of damage to the patellar tendon [9].

Surgical excision can place the skin overlying the knee at risk of vascular compromise as the anterior wall of the bursa is usually adherent to the skin. One way to reduce this risk is to excise posterior wall only but risk of recurrence still remains [10].

We present a successful 2-stage open technique using negative pressure dressings for a large chronic prepatellar bursitis.

## 2. Case Report

**Figure 1:**
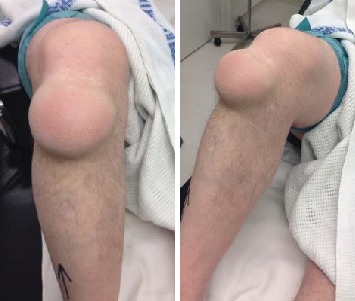
Lesion.

A 62-year-old joiner presented with 2-year history of a painless swelling over his right knee. It was occasionally tender to kneel on; however, it was the large size of the lesion that was his main problem (Figure 1). It was smooth and fluctuant. He had no limitation in knee movement. Plain radiographs did not show any bony abnormality.

**Figure 2:**
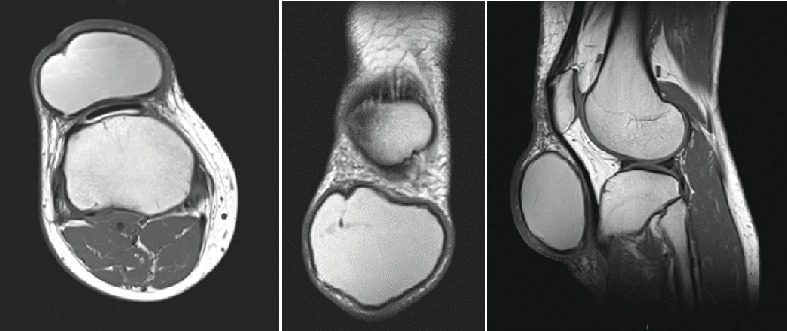
MRI scan of lesion.

A MRI scan (Figure 2) revealed a cystic lesion measuring 7.6 × 6.4 × 4.1 cm, anterior to the patellar tendon. There was no evidence of malignancy. Informed consent has been obtained from the patient to publish clinical photographs and radiological imaging.

## 3. Surgical Technique

**Figure 3:**
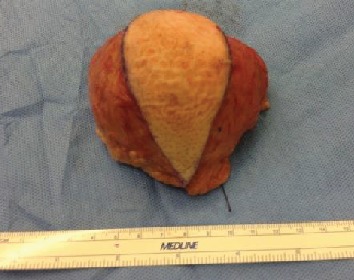
Excised lesion.

**Figure 4:**
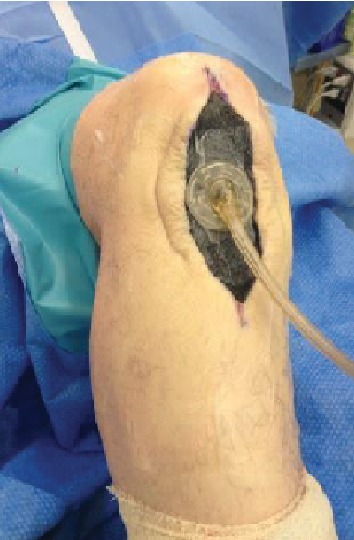
Application of VAC dressing.

**Figure 5:**
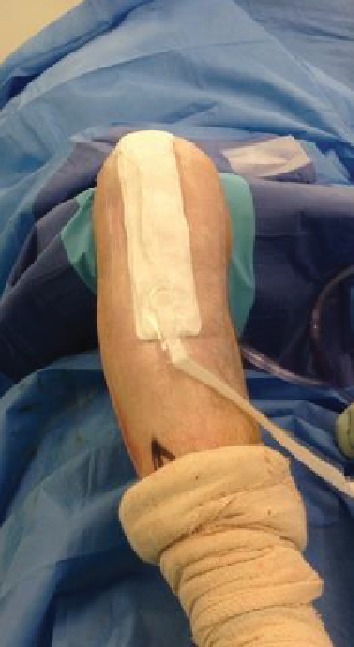
PICO dressing.

A well-circumscribed lesion was excised along with an ellipse of the skin (Figure 3). A vacuum-assisted closure (VAC) (©KCI Medical) dressing was applied (Figure 4). The patient returned to theatre after 48 hours and underwent primary closure of the wound using deep absorbable sutures to the subcutaneous layer and interrupted nylon sutures to the skin. A second negative pressure dressing, PICO© (Smith & Nephew), was applied over the wound for one week (Figure 5). He was allowed to weight bear as able and knee flexion was not restricted.

**Figure 6:**
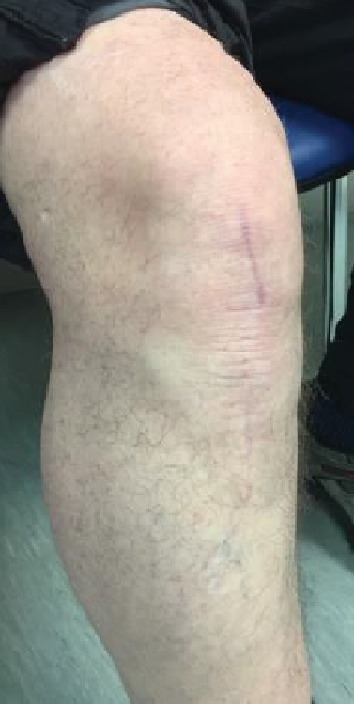
Surgical scar at 1 year.

The nylon sutures were removed at 2 weeks and he returned to work 1 week after this. He was advised not to kneel for four weeks. Histological examination confirmed a benign chronic inflammatory bursitis. He was reviewed at 1 year following his operation. His wound has healed without any complications (Figure 6). He did not have any recurrence of his bursitis, he was pain free and kneeling at work as a full-time joiner with no problems.

## 4. Discussion

Surgical management is reserved for refractory cases of prepatellar and olecranon bursae. Both open and endoscopic treatments had been described. Skin compromise is a devastating complication following surgical excision [2, 11, 12].

Negative pressure wound therapy (NPWT) has transformed management of complex wounds with its efficacy being proven extensively in the literature [13–15]. It has various beneficial mechanisms of action including reducing tissue oedema and wound tension, eliminating “dead space,” increasing wound perfusion, reducing movement at the skin edges, and possibly upregulating growth factors [16, 17]. In this case, we believe the 48 hours of NPWT created a vascular bed of granulation tissue that bonded together when primarily closed eliminating potential dead space and thus avoiding potential seroma formation and subsequent recurrence. The second negative pressure dressing (PICO) reduced the inevitable movement and increased tension about the wound when the knee flexed. This effectively splinted the wound without having to restrict knee movement.

We therefore recommend a 2-stage open surgical technique using negative pressure dressings to treat a large chronic prepatellar bursitis.
